# A Study on Pigment Composition of Buddhist Cave Paintings Based on Hyperspectral Technology

**DOI:** 10.3390/ma17215147

**Published:** 2024-10-22

**Authors:** Xiang Shi, Xiaogang Lin, Yu Lei, Jinyu Wu, Xiao Lv, Yong Zhou

**Affiliations:** 1 Key Laboratory of Optoelectronic Technology and Systems of Ministry of Education of China, Chongqing University, Chongqing 400044, China; shixiangcqu98@163.com (X.S.); 202308131171@stu.cqu.edu.cn (J.W.); a18738734288@163.com (X.L.); 2 Academy of Dazu Rock Carvings, Chongqing 402360, China; leiyu869@126.com

**Keywords:** buddhist cave paintings, high-spectrum imaging, SAM, non-destructive analysis

## Abstract

The value of the Buddhist cave lies not only in the Buddha statues but also in the surface painting. Hyperspectral imaging technology, as an emerging and effective method for component identification, offers a non-contact and non-destructive approach to the preservation and restoration of oil paintings. This study employed hyperspectral cameras to capture common pigments on the surfaces of Buddhist caves. Then, the results were processed and used as a database to identify the paintings. Additionally, a series of experiments were conducted to examine the impact of binder, substrate types, and pigment sizes on the reflectance spectrum of the paints. The Spectral Angle Matching (SAM) algorithm was then used to analyze the Yuanjue Cave and Qiqushan Stone Carvings of the Tang Dynasty in China. The findings revealed that the position of absorption peaks in the reflectance spectra is not significantly influenced by the substrate but is affected by the binder. Moreover, the absorption depth varies regularly with particle size. Furthermore, the spectral matching results demonstrate that components can be accurately identified even for similar colors. Based on the pigment distribution, the study also inferred specific details of ancient paintings, including the painting steps and hidden information in the manuscript layout. These findings hold significant implications for the restoration of representative surface paintings of the Tang Dynasty Buddhist cave, providing a reference for the selection of restoration materials and methods.

## 1. Introduction

Buddhist caves are not only outstanding representatives of ancient religious art but also vivid carriers of historical and cultural heritage. The painted sections, with their rich colors and intricate lines, showcase the exceptional skills and aesthetic pursuits of ancient artists. Furthermore, these paintings also contain rich historical and cultural information, serving as crucial materials for studying ancient society, religion, art, and cultural exchange [1]. Analyzing Buddhist cave paintings not only determines the composition of the pigments but also allows for inferring the techniques used by ancient artists through pigment distribution analysis, which provides crucial scientific data for the protection and restoration of these artworks.

Due to the unique environment of the Buddhist caves, the damage to the paintings can be categorized into natural and human factors. Natural factors include weathering, salt damage, cracking, and microbial invasion, while human factors encompass tourism activities and intentional destruction. Regardless of the type of damage, the value of the surface paintings in the caves suffers irreversible loss. Therefore, analyzing the surface paintings aims to establish preventive measures before damage occurs or to facilitate precise restoration after damage, which is crucial for preserving the value of the artifacts. Common methods for analyzing painting components include X-ray diffraction (XRD), Raman spectroscopy, X-ray fluorescence (XRF), scanning electron microscopy (SEM), Fourier-transform infrared spectroscopy (FTIR), and gas chromatography–mass spectrometry (GC-MS). The painted pigments on the grotto surfaces, having undergone historical transformations, are mostly stable mineral pigments currently detectable. A review of commonly used analytical methods is presented in Table 1, indicating that Fourier-transform infrared spectroscopy and gas chromatography–mass spectrometry are typically employed for the analysis of organic pigments, making them unsuitable for the analysis of cave painting pigments. Currently, the more established methods in the field of ancient pigment analysis involve the combination of multiple techniques, offering a more comprehensive analysis. Given that paintings are immovable, non-renewable, and cover large areas, techniques such as XRD and Raman spectroscopy do not demonstrate significant advantages. Mona F. Ali [2] utilized XRD, SEM-EDX, FTIR, and Raman spectroscopy to further investigate pigment analysis in the Iwrakhy/Hatia tomb, providing a scientific basis for understanding the preservation and restoration of ancient Egyptian wall paintings.

Techniques such as digital scanning and X-ray fluorescence spectroscopy play a crucial role in analyzing the composition of paintings. Although significant progress has been made in current painting composition analysis, there are still limitations and issues that need to be addressed. For instance, techniques like digital scanning and X-ray fluorescence spectroscopy often require localized sampling, making it challenging to achieve non-destructive analysis of the entire painting surface [9].

**Figure 1:**
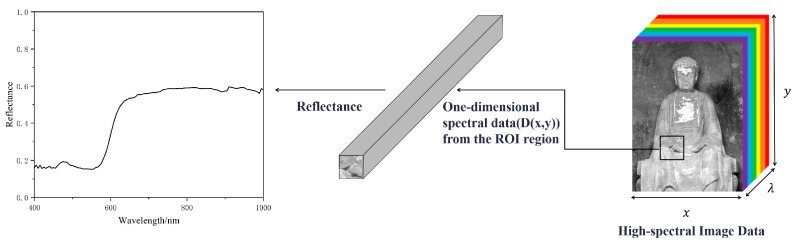
Diagram of hyperspectral data composition.

In recent years, some new technologies have been applied in the field of cultural relic composition analysis. Among these, hyperspectral imaging, as a revolutionary non-destructive detection technology, has demonstrated immense potential in artifact preservation and research. Hyperspectral imaging originated from remote sensing technology in the 1980s and has been widely applied in fields such as mineral exploration, geological analysis, food engineering, and agriculture due to its non-contact, non-destructive, and rapid capability for large-scale detection [10,11]. This technology captures optical information from continuous and narrow spectral bands reflected or emitted by the surface of objects, enabling precise measurements across a wide range of wavelengths from visible light to infrared and ultraviolet. By analyzing the generated “hypercube” dataset (Figure 1) that contains rich spectral and spatial information, it is possible to analyze the composition of materials within the region of interest (ROI) and uncover hidden information.

By analyzing the reflected spectra within hyperspectral images, this technology can effectively identify the types of pigments used in Buddhist cave paintings, providing new perspectives and methods for artifact preservation and research. Since Baronti first applied imaging spectroscopy combining visible and infrared bands to successfully extract and analyze pigment composition information from the oil painting Holy Trinity Predella in the Uffizi Gallery, Florence, in 1998, hyperspectral technology has gradually established its critical role in the field of pigment composition analysis of artworks [12]. Numerous scholars, both domestic and international, such as Casini [13], Balas [14], Cloutis [15], Li Dehui [16], Wang Lele [17], and Zhao Xinchun [18], have subsequently employed hyperspectral remote sensing technology to analyze pigment composition. These studies have consistently demonstrated that the technology can not only efficiently distinguish and identify various pigment components but also achieve the superior effect of “image-spectral fusion” while preserving image information, which provides unprecedented support for the protection and research of artworks. Furthermore, hyperspectral technology has the ability to penetrate materials and reveal underlying information. Athina Alexopoulou [19] revealed hidden underdrawings and painting layers in their study of specific oil paintings and paper sketches. Andronache [20] mentioned that combining complexity measurement with hierarchical clustering analysis can effectively identify overlapping artworks, providing important theoretical support for our understanding of overlapping layers. These studies demonstrate the potential of near-infrared spectroscopy in revealing the creative processes of artworks, especially in cases of complex layering.

Due to the complexity of data processing, equipment costs and availability, lack of standardization, and environmental influences, hyperspectral imaging technology remains in the early stages of development. So far, the application of hyperspectral imaging technology in the field of cultural heritage has primarily focused on pigment analysis of paintings and manuscripts [21], restoration of painted stone surfaces [22], damage identification [23], and extraction of hidden information [24]. There is a lack of outdoor applications of hyperspectral imaging for investigating immovable surfaces in expansive environments or open spaces, such as churches, ceilings, cloisters, or archaeological sites, as well as large-scale murals and painted surfaces. In the future, stricter standards and protocols will be established to ensure the effectiveness and repeatability of HSI in cultural heritage research, and HSI will be integrated with other technologies (XRD, 3D scanning) to improve analytical accuracy and efficiency.

Current research on pigment composition is largely based on established pigment spectral libraries, such as the Italian IFAC pigment spectral library and the USGS database. However, pigment compositions vary across different regions, making it crucial to construct and utilize an accurate pigment database for the analysis of Chinese painting pigments. Additionally, efforts on pigment, such as binders, particle sizes, and substrate materials, are still insufficient. All these factors significantly impact the spectral characteristics and stability of pigments and are key to understanding the preservation state and historical changes of painting artworks.

This study aims to construct an accurate hyperspectral database for pigments and investigate the specific impacts of binders, particle sizes, and substrate materials on pigment spectra with the aid of commonly used spectral matching algorithms in terms of composition recognition accuracy, so as to enable more precise applications of hyperspectral technology in the field of pigment composition analysis.

## 2. Materials and Methods

### 2.1. Sample Information

**Figure 2:**
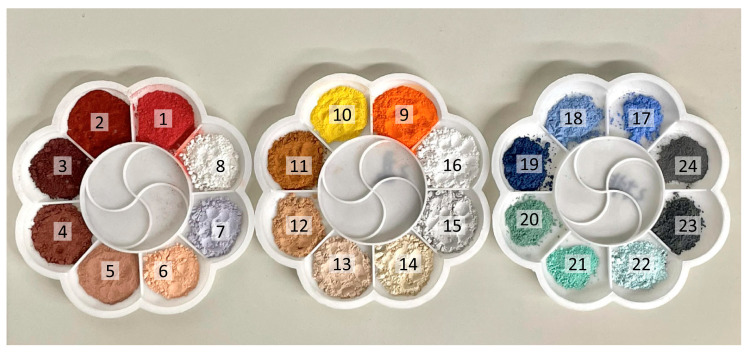
The employed mineral pigments for Chinese painting.

In collaboration with experts from the Dazu Rock Carvings, we have identified 24 common types of mineral pigments as the focus of our study. Compared to the commonly used IFAC spectral database, we have supplemented the range of ancient Chinese mineral pigments, covering colors such as red, white, yellow, blue, green, black, and others (Table 2 and Figure 2) [25].

- Red pigments include cinnabar (1), ochre red (2), iron oxide red (3), and mudstone red (5), which are commonly used for depicting facial features and clothing.
- White pigments include clam meal (8) and crystal (16). Clam meal is derived from the shells of marine clams, processed through special techniques, and commonly used for underpainting and facial features.
- Yellow pigments comprise realgar (9), orpiment (10), desert tan (11), and tea yellow (12), which are mostly symbiotic minerals and can be effectively distinguished based on variations in their color depth.
- Blue pigments mainly include lazurite (17), ultramarine blue (18), and blue flower (19). Natural ultramarine blue, also known as lapis lazuli, is an ancient mineral pigment that typically appears deep blue or bluish-green.
- Green pigment focuses on silicon malachite (20), malachite green (21), and turquoise green (22), known for their cool tones. As natural minerals, they are often used for delicate depictions of plant leaves or to create a serene landscape of mountains and rivers.
- Black pigments include blackstone (15), tourmaline (23), and ceruse (24), renowned for their deep black color and commonly used for underdrawing lines and other detailed aspects.
- Additional pigments commonly used in painting exhibit other colors, such as rock gold tea color (4), coral pink (6), purple cloud color (7), agate color (13), and coral color (14).

**Figure 3:**
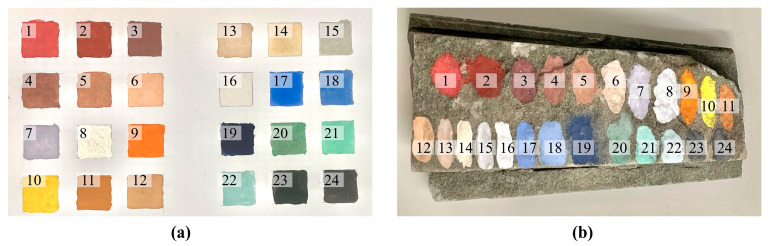
(**a**) The powder and glue are mixed and applied to the cardboard; (**b**) the powder and glue are mixed and applied to the stone.

Glue, as a key binder, not only affects the artistic expression of the painting but also directly relates to the duration that pigments remain on the substrate’s surface. As pigment powders lack adhesive properties, it is often necessary to mix an adhesive with the pigments to enhance their adhesion during the painting process. The most commonly used binder in ancient Chinese painting is animal glue. Usually, animal glue and pigment powder are mixed in a 1:1 ratio and evenly applied to industrial cardboard (Figure 3a).

Cave paintings are typically created directly on stone surfaces. In the laboratory, the methods for establishing a mineral pigment database typically include collecting the original spectra of powdered pigments or the spectra of a homogeneous mixture of powdered pigments and a binder applied to the surface of cardboard. To investigate the effect of different substrate materials on the pigment spectra, cardboard and stone substrates from the demonstration site were selected as the two experimental groups (Figure 3b).

**Figure 4:**
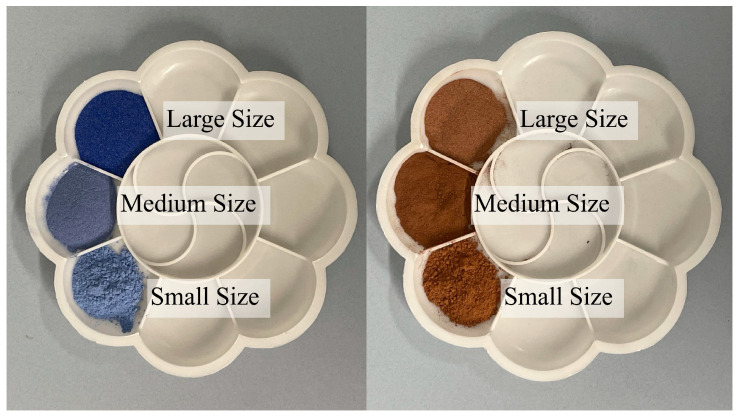
Blue flower (**left**) and ochre red (**right**) in different sizes.

The differences in pigment color are not only related to its mineral composition and oxidation state but also to the particle size of the pigment. To enrich the color gradient, artists often use pigments with different particle sizes. Blue flower and ochre red were selected to explore the relationship between particle size variation and reflectance spectra (Figure 4).

### 2.2. Data Acquisition

#### 2.2.1. High-Spectral-Resolution Imaging System

**Figure 5:**
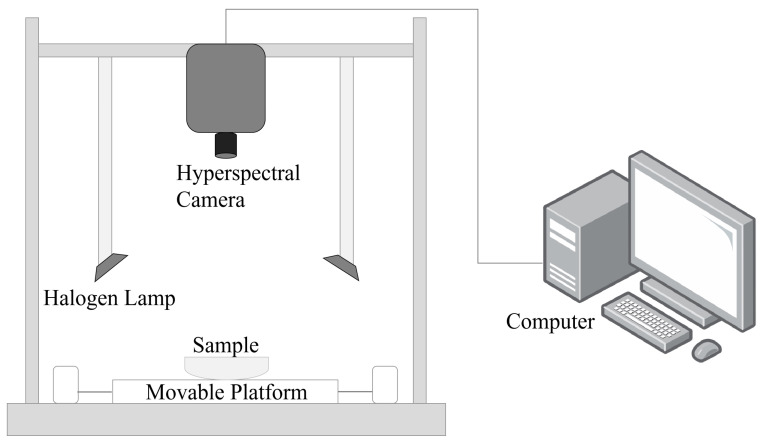
Hyperspectral data acquisition system.

The hyperspectral data acquisition system in this paper consists of three core components (Figure 5): a high-precision hyperspectral camera (SHIS Series N220 Series of Zhongda Ruihe in Shenzhen, China), a halogen lamp illumination module (with stable emission power suitable for reflectance spectrum measurement), and a high-performance computer (equipped with data acquisition software). This system enables hyperspectral imaging of mineral pigment samples in the visible to short-wave infrared spectrum (400 nm to 1000 nm).

During data collection, the hyperspectral camera needs to be preheated to ensure stable performance. This was followed by adjusting parameter settings, precise focusing, and calibration using a standard whiteboard to improve the accuracy and reliability of image acquisition. Finally, the sample to be tested was placed on a movable platform for imaging. After data collection is complete, the hyperspectral images can be viewed and saved using a host computer.

#### 2.2.2. Image Rectification

Due to the influence of the camera and the environment, hyperspectral images may contain noise or distortion. Therefore, reflectance calibration is necessary after obtaining the original hyperspectral images. Reflectance calibration requires acquiring background spectral images and instrument noise data (dark current data). A uniformly surfaced, high-reflectivity diffuse whiteboard was used to fill the entire field of view of the camera to acquire the background spectral image. A non-reflective, opaque black cover was placed over the camera lens to record an image and obtain the dark current data [26]. The relative reflectance of the original hyperspectral image, based on the region of interest pixels, was determined using Equation (1) as follows:

(1) $$R=\frac{I_{O}-I_{D}}{I_{W}-I_{D}}$$

(2) $$A=-\mathrm{lg}\left(\frac{I_{O}-I_{D}}{I_{W}-I_{D}}\right)$$

where $I_{O}$ is the raw reflectance image data, $I_{D}$ is the reflectance under dark conditions, $I_{W}$ is the pristine reflectance of the whiteboard, and R is the relative reflectance of the image. The corrected hyperspectral image is represented using absorbance (Equation (2)).

### 2.3. Analytical Methods

#### Envelope Removal

Envelope removal aims to significantly enhance the absorption trough features in the spectrum while ensuring the integrity of key information, such as the positions of sensitive bands [27]. A baseline is established using the maximum value in the spectral data to construct an envelope that fully covers the original spectral curve. This envelope is then subtracted from the original spectrum to achieve standardized data processing. The calculation methods are shown in Equations (3) and (4):

(3) $$R_{\mathrm{cn}}=\frac{R_{n}}{R_{\mathrm{start}}+K\cdot \left(\lambda_{n}-\lambda_{\mathrm{start}}\right)}$$

(4) $$K=\frac{R_{\mathrm{end}}-R_{\mathrm{start}}}{\lambda_{\mathrm{end}}-\lambda_{\mathrm{start}}}$$

$R_{\mathrm{cn}}$ represents the envelope removal value for band n, $\lambda_{n}$ refers to band n, $R_{n}$ is the original spectral reflectance for band n, $R_{\mathrm{start}}$ and $R_{\mathrm{end}}$ are the original spectral reflectances at the start and end points of the absorption curve, $\lambda_{\mathrm{end}}$ and $\lambda_{\mathrm{start}}$ are the wavelengths at the start and end points of the absorption curve, and K is the slope between the starting and ending bands. After removing the envelope, the reflectance at each point on the new spectral curve is denoted as $\rho_{c}\left(\lambda \right)$, and the spectral absorption depth at each point is calculated as:

(5) $$D_{c}\left(\lambda \right)=1-\rho_{c}\left(\lambda \right)$$

This technology eliminates background noise and interference from non-target signals, allowing for clearer identification and quantification of key absorption features in spectra. Consequently, it provides a more robust data foundation for subsequent applications such as spectral analysis, substance identification, and classification.

## 3. Results

### 3.1. Pigment Powder Spectrum

**Figure 6:**
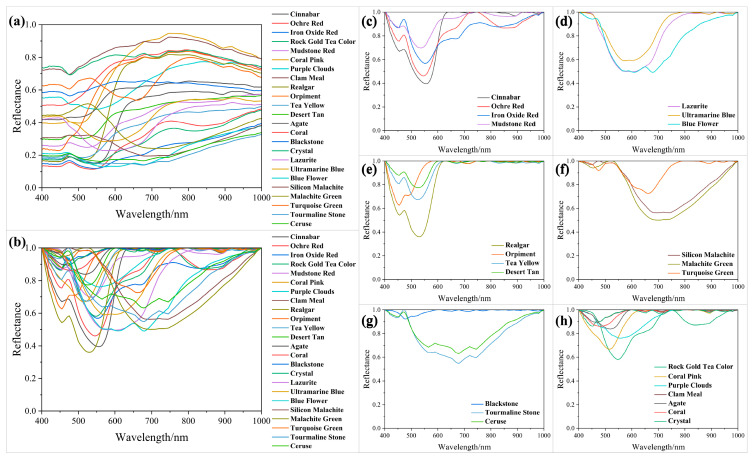
(**a**) Original spectral curve of pigment powder; (**b**) spectral curve of pigment powder after removing the envelope; (**c**) red spectrum; (**d**) blue spectrum; (**e**) yellow spectrum; (**f**) green spectrum; (**g**) black spectrum; (**h**) white and other series spectrum.

Figure 6a shows the reflectance spectra of 24 colors of mineral pigment powder obtained after data correction of hyperspectral images. Samples were drawn separately according to different color systems in order to facilitate differentiation (Figure 6b–h). At the beginning of the spectral bands between 400 and 500 nm, significant noise appears due to the reduced sensitivity of the camera’s sensor in edge bands. Because noise may obscure characteristic absorption peaks, the Savitzky–Golay convolution method was used to smooth the images. Additionally, there are notable differences among the pigment curves, with differences in reflectance and absorption peaks. Some pigments exhibit a significant increase in reflectance between 500 and 600 nm, after which the reflectance levels off.

To more clearly highlight the absorption and reflection characteristics in the spectral curves, the collected mineral pigment spectra underwent envelope removal processing. This treatment not only enhances the absorption trough features but also preserves the integrity of key information, such as the positions of sensitive bands (Figure 6b).

### 3.2. Influence of Gluing on Spectrum

**Figure 7:**
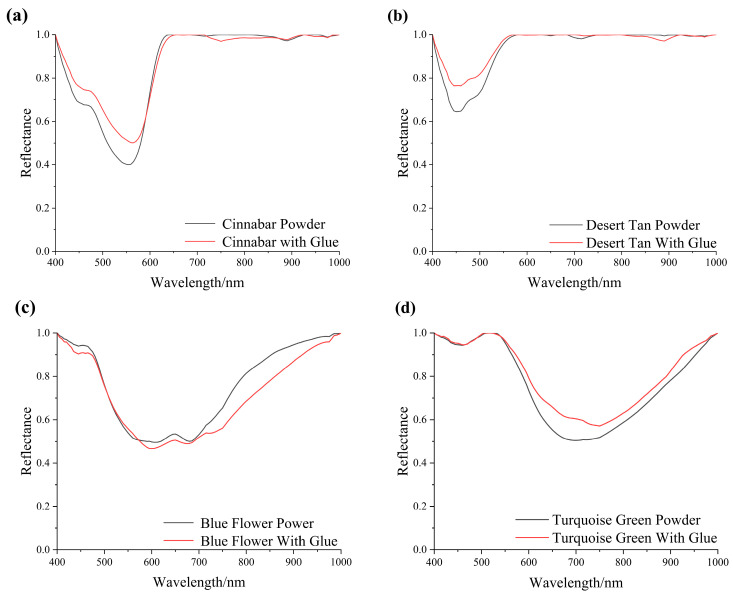
Spectral comparison of powder and powder mixed glue: (**a**) cinnabar; (**b**) desert tan color; (**c**) blue flower color; (**d**) turquoise green color.

Representative colors from four common hues (red, yellow, blue, and green) were selected (Figure 7). As shown in Figure 7a, there is a significant difference in emissivity between powdered cinnabar and cinnabar mixed with glue within the range of 400 to 580 nm. At 750 nm, cinnabar with glue forms a small absorption peak. For desert tan (Figure 7b), there is a difference in reflectance between 400 and 550 nm. At 700 nm, desert tan powder shows a small absorption peak, whereas the spectrum with glue does not. Conversely, the spectrum with added glue exhibits a new absorption peak at 900 nm. The spectra remain overlapping at other wavelengths. The spectral curves of blue flower overlap from 400 to 700 nm (Figure 7c), with small differences noted beyond 700 nm. The spectral curve of turquoise green completely overlaps between 400 and 550 nm, with slight differences thereafter (Figure 7d).

Overall, the spectral emissivity of the glued and powder samples exhibits a generally similar pattern. Notably, the influence of the adhesive can lead to the loss of original absorption peaks and the formation of new ones. Therefore, incorporating glued spectra into the original spectral library is expected to significantly enhance identification accuracy.

### 3.3. Influence of the Substrate Material

**Figure 8:**
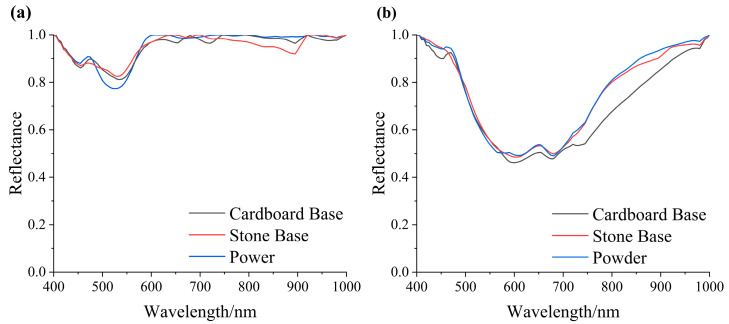
Spectrograms of pigments based on different substrates: (**a**) desert tan color and (**b**) blue flower color.

Taking desert tan and blue flower as examples, the reflectance spectra indicate that the absorption feature positions for powder, cardboard, and stone substrates are essentially consistent. At 500 nm, there is a weak absorption peak for desert tan, and at 600 nm to 700 nm, there is a broader absorption peak for blue flower (Figure 8). There are slight differences in the absorption depth among the different substrates. The reason is that, compared to the powder, the spectral curves for the color card and stone substrates are obtained after applying a 1:1 mixture of pigment and gelatin on the surface. Since the pigment thickness is thin and easily penetrated, and the color of the color card substrate is light, while that of the rock substrate is dark, the reflectance is affected, leading to changes. Overall, this factor has a small impact when establishing a standard pigment spectral library.

### 3.4. Influence of the Particle Size

**Figure 9:**
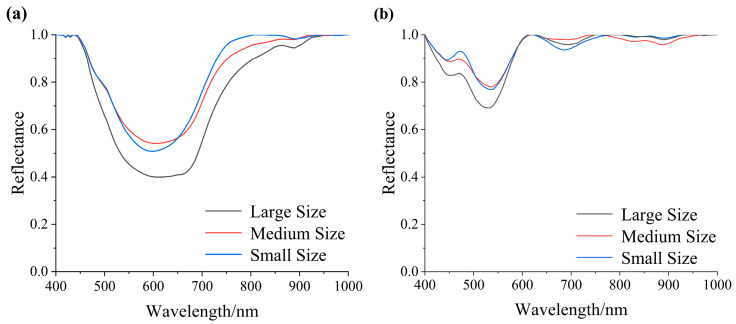
Spectra of different particle sizes: (**a**) lazurite and (**b**) ochre red.

In order to enrich the color gradations of the paintings, painters use grinding techniques to classify mineral pigments into different particle size grades. The pigments of different particle size grades exhibit different hues, thereby enhancing the color expression and artistic value of the paintings. Taking lazurite and ochre red as an example, three particle sizes were selected for comparison: large (100 μm), medium (50 μm), and small (30 μm) (Figure 9).

The spectral curves of lazurite with different particle sizes show a consistent overall trend, the reflectance value decreases first and then increases, forming a broad absorption feature peak in the 550 nm to 650 nm range, with a relatively flat variation after 800 nm. The reflectance value of the spectral curves of ochre red first decreases, then slightly rebounds, and subsequently continues to decline near 600 nm, rising to 1. The depth of the absorption peaks shows a systematic difference; larger particle-size pigments have greater absorption depths, while smaller particle-size pigments exhibit shallower absorption. Therefore, to achieve more accurate spectral recognition, it is essential to consider the particle size when selecting recognition algorithms.

### 3.5. Spectrum Matching

We organized the spectra of the powdered pigments, pigments with binders, and pigments with different particle sizes from the above 24 mineral pigments to build a database for subsequent spectral matching and identification. Compared to other databases, this one comprehensively considers the various factors affecting the painting process, serving as a targeted dataset for studying Chinese Buddhist grotto murals and making it possible to improve the efficiency and accuracy of spectral matching. To evaluate the effectiveness of spectral matching algorithms such as minimum distance matching, correlation coefficient matching, information divergence matching, spectral angle matching, and encoding matching [28,29,30], 100 groups of similar spectral samples and 100 groups of dissimilar spectral samples were set up. The same method was used to match similar material spectra, and the average match value was calculated. Similarly, the average match value for dissimilar material spectra was computed. The matching effectiveness was determined based on the ratio of these values; a smaller ratio indicates a better match. As shown in the table below, the spectral angle matching method achieved the best experimental results, followed by information divergence matching and correlation coefficient matching (Table 3).

#### Spectral Angle Matching Algorithm

**Figure 10:**
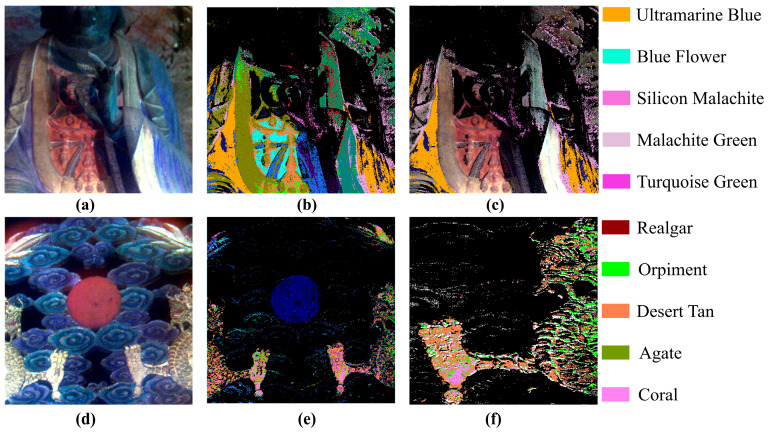
Spectrum identification results: (**a**) Dazu Rock Carvings; (**b**) overall recognition result; (**c**) results of sleeve pigment recognition; (**d**) Qiqu Mountain Grottoes; (**e**) overall recognition result; (**f**) local detail of the dragon on the right.

The Spectral Angle Matching (SAM) algorithm is a method for determining the similarity between two spectral curves based on the numerical value of the angle between the target spectrum and the test spectrum. The smaller the angle between two spectra, the higher the degree of matching, where θ represents the angle between the two spectra:

(6) $$\mathrm{SAM}\left(x,y\right)=\cos^{-1}\frac{\sum \mathrm{xy}}{\sqrt{\sum {\left(x\right)}^{2}\sum {\left(y\right)}^{2}}}$$

x and y represent the spectral curves of the reference spectrum and the test spectrum, respectively. The magnitude of the spectral angle depends solely on the direction of the two compared spectral vectors and is independent of their radiance, thereby reducing the influence of illumination and terrain on the similarity measurement. This offers advantages in spectral recognition affected by variations in reflectance due to particle size [31,32]. Therefore, this paper’s spectral recognition is based on the spectral angle mapping algorithm, with the angle set to 0.1, using the organized pigment spectral library as the training set to identify and classify the murals of the Dazu Rock Carvings and the Qiqu Mountain Grottoes (Figure 10).

The World Cultural Heritage site of Dazu Stone Carvings in Chongqing represents the highest level of stone cave art from the 9th to the 13th centuries. The samples selected for this article come from the Beishan Stone Carving Group in Dazu, which began construction in the Jingfu Year of the Tang Dynasty, 892 AD, and continued through the periods of the Front and Rear Sichuan Kingdoms, ending around 1162 AD with the completion of large-scale sculpture activities during the Southern Song Dynasty. The same colors map the areas in the cave murals where the same pigment is used, while areas where the pigments could not be clearly identified are marked in black. Visual observation of the original image reveals that the colors of the Buddha’s sleeves are primarily concentrated in the blue and green ranges (Figure 10a). Identification of the sleeve area was conducted using the blue and green standard pigment spectral libraries as input, and the results are shown in Figure 10c, indicating that the sleeve is composed of ultramarine blue, blue flower, silicon malachite, malachite green, and turquoise green. The color distribution effectively validates the consistency between actual observations and spectral identification conclusions, thus demonstrating the reliability of hyperspectral imaging in the analysis of painted pigment compositions.

The stone carvings on Qiqushan Mountain show the superb skills of the artists of the same period in the Tang Dynasty. Figure 10d presents the representative painting on the Qiqu Mountain Buddhist Grottoe, where the symmetrically depicted dragons on both sides exhibit a unified yellow tone. However, the results of spectral analysis indicate that the color composition of the dragons is not singular (Figure 10f), displaying a patterned color distribution. Specifically, the main framework of the dragon is painted in orpiment, while the blank areas between the framework are filled with desert tan, enhancing the transitional effect of the colors and the artistic expressiveness of the painting. The skeletal structure of the dragon is distinctly observable, enabling us to deduce the framework employed by the artist during the painting process. Additionally, the dragon’s feet cleverly incorporate coral and agate colors, enriching the layers of the artwork and providing a reference for accurately restoring the dragon’s details.

The experimental results show that high-spectrum imaging technology can achieve non-destructive, large-scale, detailed analysis of the pigments used in Buddhist cave paintings, allowing for a more accurate recording and understanding of the types of pigments used. Even subtle color differences that are difficult to distinguish with the naked eye due to natural weathering can be clearly displayed in high-spectrum image analysis, helping us choose the most appropriate colors during restoration to better restore the paintings. The regular color distribution of the dragon pigment used in the painting not only provides researchers with clues to infer the design and painting process of the original painting but also proves that high-spectrum imaging technology can open up new paths and methods for in-depth exploration of the inherent information of paintings and other cultural relics, and assist in the identification of cultural relics.

## 4. Conclusions

This study is dedicated to investigating the compositional structure of surface paintings in Buddhist caves. Using envelope removal processing technology, a standard library of painting pigments was successfully constructed. Taking into account the characteristics of ancient painting techniques, the specific effects of binder addition and particle size on pigment imaging spectra were systematically analyzed. Using the Spectral Angle Mapper (SAM) algorithm, spectral matching analysis was performed on the Buddhist cave paintings of the Dazu Rock Carvings—Yuanjue Cave and representative paintings at Qiqu Mountain. This enabled the accurate identification of painting pigments and the effective extraction of hidden information.

The results indicate that different substrates have minimal impact on the overall trend of pigment reflectance spectra in the visible and near-infrared bands, whereas the addition of glue and different particle sizes do affect the spectra. Therefore, it can be considered to include glued pigments as part of the standard library, providing a convenient and accurate reference for subsequent studies on painting compositions. The SAM algorithm performs excellently in pigment recognition, effectively distinguishing closely related colors that are difficult to differentiate with the naked eye. Analyzing the identified results allows for inferences about the artistic techniques and some hidden information employed by the artist during the painting process.

In summary, this research leverages hyperspectral technology to investigate the composition of Buddhist cave paintings, offering a scientific foundation for exploring the intrinsic value of cultural relics. It introduces a non-destructive, efficient, and precise methodology for relic identification and conservation, holding significant theoretical and practical implications. This work is poised to advance relic research and conservation, contributing substantially to the protection and preservation of cultural heritage in the future.

## Figures and Tables

**Table 1:** Comparison of commonly used methods for composition analysis of pigments.

Method	Principle	Advantages	Disadvantages	Applicable Range	Ref
XRD	Analyzes the crystal structure of materials using X-ray diffraction	High sensitivity, non-destructive	Can only analyze crystalline samples	Qualitative and quantitative analysis of crystalline pigments	[3]
SEM	Scans the surface of the sample with an electron beam to observe morphology and composition	High-resolution surface morphology images	Requires conductive samples, complex sample preparation	Morphology and microstructure analysis of pigments	[3]
FTIR	Analyzes molecular vibrations using infrared spectroscopy	High sensitivity, identify organic compounds	Sensitive to moisture, requires sample treatment	Functional group analysis of organic pigments	[4]
GC-MS	Combines gas chromatography and mass spectrometry to separate and identify compounds	High sensitivity and resolution	Complex samples require preparation, expensive equipment	Component analysis of organic pigments	[5]
XRF	Analyzes elemental composition by exciting the sample with X-rays to emit characteristic X-rays	Fast, non-destructive, can detect multiple elements simultaneously	High cost, limited detection of light elements, interference with substrate materials	Elemental composition analysis of inorganic pigments	[6]
Raman spectroscopy	Analyzes molecular vibrations based on Raman scattering	No sample preparation needed, suitable for liquids and solids	Sensitive to fluorescence interference	Molecular structure analysis of organic and inorganic pigments	[7]
HSI	Captures spectral information across multiple bands	Non-destructive, high-resolution, rich spectral information, large detection area.	High cost, complex data processing	Distribution and composition analysis of a wide range of pigments	[8]

**Table 2:** Mineral pigments used for Chinese painting.

Color	Elements	Purpose
Red (1, 2, 3, 5)	HgS, $S,\;{\mathrm{Fe}}_{2}O_{3}$	Faces and costumes
White (8, 16)	${\mathrm{CaCO}}_{3}$, ${\mathrm{SiO}}_{2}$	Manuscript and faces
Yellow (9, 10, 11, 12)	${\mathrm{Fe}}_{2}O_{3}\cdot 3H_{2}O$, AsS, ${\mathrm{As}}_{2}S_{3}$	Dress and decorative patterns
Blue (17, 18, 19)	$2{\mathrm{CuCO}}_{3}\cdot \mathrm{Cu}{\left(\mathrm{OH}\right)}_{2}$, ${\left(\mathrm{Na},\mathrm{Ca}\right)}_{4-8}{\left({\mathrm{AlSiO}}_{4}\right)}_{6}{\left({\mathrm{SO}}_{4},S,\mathrm{Cl}\right)}_{1-2}$	Nature and fantasy
Green (20, 21, 22)	${\mathrm{CuCO}}_{3}\cdot \mathrm{Cu}{\left(\mathrm{OH}\right)}_{2}$, ${\mathrm{Cu}}_{2}{\left(\mathrm{OH}\right)}_{2}{\mathrm{CO}}_{3}$	Dress and nature
Black (15, 23, 24)	C	Outline and Architecture

**Table 3:** Spectral matching results.

Matching Method	Homogeneous Matching Mean	Heterogeneous Matching Means	Ratio of Difference
Absolute value distance method	1.92647	3.34092	0.57663
Ming distance method	1.32578	2.83578	0.46752
Tangent distance method	2.83972	4.35497	0.65206
Correlation coefficient method	0.92736	0.16835	0.18154
Information divergence method	0.01732	0.32459	0.05336
Spectral Angle method	0.91691	0.03135	0.03419
Code matching method	0.92567	1.92384	0.48116

## Data Availability

The original contributions presented in the study are included in the article, further inquiries can be directed to the corresponding authors.
